# Validation of measurement of body composition by dual-energy X-ray absorptiometry and bioelectrical impedance analysis and body composition’s profiling in Tibetan’s adults

**DOI:** 10.1017/S1368980025000291

**Published:** 2025-04-28

**Authors:** Wenxiu Jian, Bin Zhang, Yue Ma, Xiao Tang, Tuan Thanh Nguyen, Meng Lv, Xiangyang Meng, Tiemei Li, Xiaomin Sun, Youfa Wang, Yanming Ren, Wen Peng

**Affiliations:** 1 Nutrition and Health Promotion Center, Department of Public Health, Medical College, Qinghai University, Xining, China; 2 School of Mathematics and Statistics, Qinghai Minzu University, Xining, China; 3 International Obesity and Metabolic Disease Research Center, Global Health Institute, School of Public Health, Xi’an Jiaotong University, Xi’an, China; 4 Alive & Thrive, FHI 360, Hanoi, Vietnam; 5 Medical College, Qinghai University, Xining, China; 6 Qinghai Provincial Key Laboratory of Prevention and Control of Glucolipid Metabolic Diseases with Traditional Chinese Medicine, Xining, China

**Keywords:** Body composition, DXA, Bioelectrical impedance, Validation, Tibetan

## Abstract

**Objective:**

We aimed to validate in-body bioelectrical impedance analysis (BIA) measures with dual-energy X-ray absorptiometry (DXA) as reference and describe the body composition (BC) profiling of Tibetan adults.

**Design:**

This cross-sectional study included 855 participants (391 men and 464 women). Correlation and Bland–Altman analyses were performed for method agreement of in-body BIA and DXA. BC were described by obesity and metabolic status.

**Setting:**

In-body BIA and DXA have not been employed to characterise the BC of the Tibetan population living in the Qinghai–Tibet Plateau.

**Participants:**

A total of 855 Tibetan adults, including 391 men and 464 women, were enrolled in the study.

**Results:**

Concordance correlation coefficient for total fat mass (FM) and total lean mass (LM) between in-body BIA and DXA were 0·91 and 0·89. The bias of in-body BIA for percentages of total FM and total LM was 0·91 % (2·46 %) and –1·74 % (–2·80 %) compared with DXA, respectively. Absolute limits of agreement were wider for total FM in obese men and women and for total LM in overweight men than their counterparts. Gradience in the distribution of total and regional FM content was observed across different BMI categories and its combinations with waist circumference and metabolic status.

**Conclusions:**

In-body BIA and DXA provided overall good agreement at the group level in Tibetan adults, but the agreement was inferior in participants being overweight or obese.

Assessment of body composition (BC) is considered an alternative and perhaps more precise approach for identifying adiposity and predicting CVD^(1,2)^, given the heterogeneity in the association of adiposity measured by BMI with CVD^(3,4)^. Various methodologies are available for assessing BC, including dual-energy X-ray absorptiometry (DXA) and in-body bioelectrical impedance analysis (BIA). While DXA is considered more accurate and a gold standard for BC measurement, it is accompanied by cost implications and operational complexities. In contrast, in-body BIA presents a more convenient option with fewer logistical challenges^(5)^. Some studies have validated the concurrence of in-body BIA and DXA in BC assessment in populations primarily living in well-developed cities^(6–8)^. However, such studies are scarce in populations living in remote and resource-limited areas.

Adiposity in the Tibetan population living in high-altitude areas is a very interesting but seldom studied research topic, as well as an important public health issue. National surveillance data in China in 2013–2014 showed prevalent central obesity (CO) but a low level of obesity prevalence in the Tibetan Autonomous region, where almost 90 % residents are of Tibetan ethnicity^(9)^. Specifically, the prevalence of CO, measured by waist circumference, was 27–34 % in men and 40–55 % in women, while the prevalence of obesity, measured by BMI, was only 4–9 % in both men and women^(9)^. This inconsistent finding suggested that the BC profiling of the Tibetan population may be quite differently from other populations, which is shaped by the unique hypobaric and extreme cold environment and distinct traditional subsistence and lifestyles in the high-altitude plateau^(10)^. On the other hand, our previous studies have shown increasing and high prevalence of obesity, and the combined prevalence of overweight and obesity reached 47·9 % among the Tibetan population^(11,12)^. This was probably associated with the highest level of mortality rates from CVD in the Tibetan population in China^(13)^.

In-body BIA is a more practical approach for BC measurement for population study among Tibetan compared with DXA, given the challenges in using DXA brought by the remoteness and inferior infrastructure of Tibetans’ residing sites. However, validation of in-body BIA method against DXA is needed specifically for the Tibetan population because of their unique BC mentioned above. Despite many validation studies across populations^(7,14)^, we did not find an independent study among the Tibetan population, which measured BC using DXA method, let alone validate in-body BIA measurement in assessing BC by using DXA as a reference.

To address the research gap, this study aimed to (1) validate the concordance between DXA and in-body BIA techniques in measuring BC and (2) describe the BC attributes among Tibetan adults living in the Qinghai-Tibet Plateau.

## Materials and methods

### Participants

Participants were recruited from two settled Tibetan communities in the suburb of Golmud City (2800 m above sea level). The inclusion criteria were (i) Tibetan adults aged ≥ 18 years; (ii) having lived in one of the two surveyed communities for more than 3 years; (iii) being able to complete the questionnaire (face-to-face) and assessments and (iv) being willing to participate in this study and giving full informed consent for inclusion before the study. The exclusion criteria were (i) pregnant women; (ii) severe physical or mental illness; (iii) standard exclusions for DXA or in-body BIA: (a) weight ≥ 204 kg or height ≥ 197·5 cm; (b) currently pregnant or planning to become pregnant; (c) presence of limb amputations, scoliosis or surgical implants, such as prostheses, pacemakers, stents, braces (e.g. dental braces) and other internal metallic devices and (d) intake of barium or intravenous contrast agents within the past 7 d. A total of 1611 community members were enrolled in the survey after signing an informed consent from December 2021 to May 2022. The present study included subjects who had completed anthropometric measurements and BC assessment by both DXA and in-body BIA and excluded those with missing data for the required variables. A total of 855 Tibetan adults aged 18–85 years were included in the analysis.

### Data collection

Social-demographic and lifestyle data, such as ethnicity, education, smoking status, etc., were gathered by questionnaire through a face-to-face interview by trained investigators. Height and weight were measured by trained staff using regularly calibrated, fully automated height and weight scales – Aipurui IPR-scale08 (Aipurui, China). Waist circumference was measured using a non-stretching soft tape at the midpoint between the lowest rib margin and the iliac crest^(15)^. Weight, height and waist circumference were measured in duplicate, and an averaged value of two measurements was used. The BMI was calculated by dividing height (m) by the square of body weight (kg). After resting for ≥ 15 min, blood pressure was measured by an electronic sphygmomanometer (OMRON HEM-7312, Japan) 3 times with 1- to 2-min intervals in a sitting position from the right arm using a suitable cuff size based on the arm circumference. The mean of the last two readings was used for analysis.

### *BC measurement with* dual-energy X-ray absorptiometry *and in-body BIA*

A whole-body DXA (Hologic Horizon W, USA) scan was performed to measure the total and regional body fat mass (FM), lean mass (LM) and bone mineral densitometry using the DXA technique. Each participant underwent separate scans of the lumbar spine, hip and whole body. Measurements and quality control were conducted by trained staff according to standard procedures. The specific procedures were as follows: (1) A standard phantom was checked before calibrating of the DXA machines and scanning the participants every morning; (2) Four operators at each study location were trained by the same technician certified by the International Society for Clinical Densitometry administered DXA procedures, the training materials included the International Society for Clinical Densitometry’s official technician hands-on training materials and the manufacturer’s handbook including testing procedures and operation methods; (3) The lumbar spine and hip joints of 15 participants were scanned three times for computational accuracy. After each scan, they had to leave the scanner to repose before the next scan. For formal measurements, each participant was scanned only once at each site; (4) All participants were requested to remove outer garments and objects that would potentially interfere with testing, and the volunteers were repositioned for each scan^(16)^. All DXA values were analysed using Hologic Apex software (version 4.0) following the manufacturer’s guidelines.

A BC analyser (Inbody 270, Korea) was also used for BC measurement in participants with the in-body BIA technique, with standard procedure. It utilises direct segmental multi-frequency in-body BIA with an eight-point tactile electrode method to measure BC. This method is based on measuring electrical impedance or opposition to the flow of a small alternating current applied to the body. The participants stood upright while measured, with hands holding the electrodes and feet on the electrodes, wearing light clothing with pockets emptied, no metal objects and no shoes^(17)^.

### Laboratory assay

Blood sample was collected after an overnight fasting period of at least 12 h. Metabolic indicators, such as fasting plasma glucose and HDL cholesterol, were measured by a certified laboratory in a local hospital.

### Data analysis and statistics

Values of total and regional BC (FM, LM, body fat percentage [%BF] and LM percentage) were analysed. Data were presented as mean (sd) or median (IQR) for continuous measures and frequency (percentage) for categorical measures. The bias for the absolute difference between values derived from DXA and in-body BIA was calculated by [DXA value-in-body BIA value], and the percentage of difference (%) was calculated by [100*×* 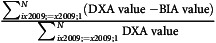 ]. To evaluate relative agreement of the two methods, Spearman’s correlation coefficient and Lin’s concordance correlation coefficient, ρ were used^(18)^. We then analysed the correlation of DXA and in-body BIA measures for trisection by kappa coefficient^(19)^. To verify the degree of agreement among the methods^(18)^, Bland–Altman analysis^(20)^ was performed to determine absolute limits of agreement between the BC variables assessed by the two methods. Spearman’s correlation coefficient between absolute difference and average of DXA and in-body BIA values was calculated in Bland–Altman analysis. Individuals from different age groups, 18–44, 45–59 and ≥ 60 years, were shown in Bland–Altman plots. Chi-square test, independent Kruskal-Wallis *H* test, independent *t*-test and Mann–Whitney *U* test were used to determine differences at group level.

The sample was analysed as a whole group and then classified into three sub-groups^(21)^: under-/normal weight (BMI < 24 kg/m^2^), overweight (BMI: 24·0–27·9 kg/m^2^) and obesity (BMI ≥ 28 kg/m^2^). Underweight individuals were analysed together with the normal group due to the small sample size. BC characteristics of participants who had CO or metabolic syndrome (MetS) were compared with those without. CO was defined as waist circumference ≥ 90 cm for men or ≥ 80 cm for women^(22)^. MetS was defined if ≥ 3 criteria were fulfilled: (1) CO; (2) fasting plasma glucose ≥ 5·6 mmol/l or on medication for high blood glucose; (3) systolic blood pressure ≥ 130 mmHg or diastolic blood pressure ≥ 85 mmHg or on antihypertensive medication; (4) HDL cholesterol < 1·03 mmol/l for men and < 1·30 mmol/l for women or on medication for reduced HDL cholesterol and (5) TAG ≥ 1·7 mmol/l or on medication for elevated TAG^(23)^. Statistical analysis was performed using Stata software version 17·0. For all analyses, two-sided *P* values of 0·05 were considered statistically significant.

## Results

### *Comparison between* dual-energy X-ray absorptiometry *and in-body* bioelectrical impedance analysis *measurements*

Data of 391 men and 464 women were analysed, among whom the average age was 47·4 ± 13·7 years, and 74·5 % have never received an education. Summary demographics of the participants included in the analysis are shown in Table 1. For the total participants, the average BMI was 27·0 ± 5·1 kg/m^2^ with a range from 14·1 to 57·8 kg/m^2^.

**Table 1:** Demographic and clinical characteristics of participants

Variables	Total		Men		Women		*P* value^¶^
	Mean or median	sd or IQR	Mean or median	sd or IQR	Mean or median	sd or IQR	
*n*, %	855	100	391	45·7	464	54·3	
Age, years	47·4	13·7	48·1	14·3	46·8	13·3	0·17
Age group, years							0·02
18–44	352	41·2	154	39·4	198	42·7	
45–59	343	40·1	148	37·9	195	42·0	
60 or older	160	18·7	89	22·8	71	15·3	
Education, *n*, %							0·01
No schooling	637	74·5	273	69·8	364	78·4	
<Primary school	66	7·7	39	10·0	27	5·8	
≥Primary school	152	17·8	79	20·2	73	15·7	
Smoking, *n*, %^*^							<0·001
Never	708	82·8	282	72·1	426	91·8	
Former smokers	24	2·8	15	3·8	9	1·9	
Current occasional smokers	19	2·2	10	2·6	9	1·9	
Current frequent smokers	104	12·2	84	21·5	20	4·3	
Alcohol consumption, *n*, %^†^							<0·001
Never	752	88·0	323	82·6	429	92·5	
Former alcohol drinkers	24	2·8	19	4·9	5	1·1	
Current occasional alcohol drinkers	69	8·1	43	11·0	26	5·6	
Current frequent alcohol drinkers	10	1·2	6	1·5	4	0·9	
BMI, kg/m^2^	27·0	5·1	26·7	4·7	27·4	5·4	0·04
Body mass status, *n*, %							0·44
BMI < 24 kg/m^2,^ *n*, %^‡^	258	30·2	124	31·7	121	28·9	
BMI: 24–27·9 kg/m^2^	245	28·7	115	29·4	130	28·0	
BMI ≥ 28 kg/m^2^	352	41·2	152	38·9	200	43·1	
Waist circumference, cm	92·3	13·0	94·2	13·2	90·6	12·6	<0·001
Central obesity, *n*, %^§^	612	71·9	248	63·6	364	79·0	<0·001
Metabolic syndrome, *n*, %^||^	339	41·5	160	42·7	179	40·5	0·53

Data are presented as mean ± sd or median (IQR) for continuous measures, and frequency (percentage) for categorical measures.

* Current occasional smokers were participants smoking less than five cigarettes/day; current frequent smokers were participants smoking more than five cigarettes/day.

† Current occasional alcohol drinkers were participants with alcohol consumption less than 40 g/week; current frequent alcohol drinkers were participants with alcohol consumption more than 40 g/week.

‡ Underweight (BMI < 18·5 kg/m^2^), *n* 21 (2·5 %); normal weight (BMI: 18·5–23·9 kg/m^2^), *n* 237 (27·7 %).

§ Central obesity was defined as a waist circumference ≥90 cm for men or ≥80 cm for women.

|| Metabolic syndrome was defined if ≥3 criteria were fulfilled: (1) central obesity; (2) fasting plasma glucose ≥ 5·6 mmol/l or on medication for high blood glucose; (3) systolic blood pressure ≥130 mmHg or diastolic blood pressure ≥85 mmHg or on antihypertensive medication; (4) HDL cholesterol (HDL-C) <1·03 mmol/l for men and <1·30 mmol/l for women or on medication for reduced HDL-C; (5) TAG ≥ 1·7 mmol/l or on medication for elevated TAG.

¶ According to *χ* ^2^ test and independent *t*-test.

The values of body FM and LM and their difference in values assessed by DXA and in-body BIA are presented as median (IQR) in Table 2. Regarding total FM in all participants, the difference between the DXA and in-body BIA values was –0·15 kg (–8·05, 7·75). As for total fat-free mass (LM), the difference between the DXA and in-body BIA values was –1·49 kg (–8·74, 5·76) (Table 2). Total fat and LM estimations showed a bias lower than 4 % for men, women and the total subjects, whereas bias for arm and leg BC measures were generally higher, with a bias for leg FM in women at 1·64 kg (17·61 %) (Table 2). The correlation of BC estimations using In-Body BIA and DXA were strong for all tested variables (*P* < 0·001) (Table 2), with the Spearman’s *r* of total FM and truncal FM measured by in-body BIA and DXA ≥ 0·90 in men, women and the total, though Lin’s *ρ* ranged from mediocre (0·66 for percentage total LM in men and arm LM in women) to very good (0·92 for total FM in women) depending on the two methods (Table 2). Kappa values also demonstrated a substantial agreement (> 0·60) between DXA and in-body BIA when dividing total FM into trisection categories in men, women and the entire sample (Table 2). However, the kappa coefficient generally showed moderate agreement with respect to the five lean body mass variables in men (Table 2).

**Table 2:** Comparison of body fat mass (FM) and lean body mass obtained by dual-energy X-ray absorptiometry (DXA) and bioelectrical impedance analysis (in-body BIA) in Tibetan adults

	DXA		BIA		Difference^*^	95 % CI	Percentage of difference (%)^†^	Spearman *r* ^‡^	Lin *ρ*	Kappa^§^
	Median	IQR	Median	IQR						
1. Total (*n* 855)										
A. Body FM, kg										
Total FM	25·31	19·62, 30·83	25·50	18·30, 31·80	–0·15	–8·05, 7·75	–0·58	0·91	0·91	0·68
Truncal FM	12·62	9·32, 16·09	13·70	9·60, 17·00	–0·66	–5·11, 3·79	–5·11	0·91	0·89	0·66
Leg FM	7·73	6·19, 9·70	7·00	5·20, 8·60	1·02	–2·49, 4·53	12·56	0·78	0·74	0·48
Arm FM	3·24	2·40, 4·19	3·50	2·30, 5·00	–0·48	–2·97, 2·01	–14·11	0·88	0·76	0·61
Percentage body fat, %	37·27	31·95, 43·25	36·70	29·50, 43·60	0·91	–9·52, 11·34	2·46	0·85	0·82	0·58
B. Lean body mass, kg										
Total lean mass	41·53	35·60, 48·36	42·90	36·80, 50·20	–1·49	–8·74, 5·76	–3·51	0·91	0·89	0·68
Truncal lean mass	20·99	18·16, 24·32	20·80	17·80, 23·90	0·33	–3·84, 4·50	1·55	0·87	0·87	0·58
Leg lean mass	12·52	10·37, 14·93	12·68	10·65, 15·35	–0·24	–3·00, 2·52	–1·88	0·9	0·88	0·70
Arm lean mass	4·56	3·65, 5·63	4·85	3·92, 5·86	–0·28	–1·34, 0·78	–6·04	0·91	0·88	0·68
Percentage lean mass, %	62·05	56·23, 67·02	63·28	56·34, 70·50	–1·74	–12·95, 9·47	–2·80	0·84	0·78	0·57
2. Men (*n* 391)										
A. Body FM, kg										
Total FM	23·09	17·24, 28·95	23·60	16·20, 30·30	–0·28	–8·47, 7·91	–1·19	0·90	0·90	0·67
Truncal FM	12·24	8·76, 15·82	13·00	8·50, 16·50	–0·18	–4·65, 4·29	–1·46	0·90	0·91	0·64
Leg FM	6·61	5·27, 8·04	6·30	4·70, 7·80	0·29	–2·67, 3·25	4·30	0·80	0·77	0·50
Arm FM	2·82	2·02, 3·60	3·00	1·80, 4·30	–0·41	–2·84, 2·02	–14·08	0·87	0·72	0·58
Percentage body fat, %	32·18	27·60, 35·77	31·50	24·80, 37·10	0·60	–10·00, 11·20	1·90	0·77	0·74	0·47
B. Lean body mass, kg										
Total lean mass	48·64	44·60, 53·01	50·50	46·10, 55·50	–1·65	–9·49, 6·19	–3·37	0·83	0·82	0·51
Truncal lean mass	24·13	21·78, 26·67	24·00	21·90, 26·60	0·22	–4·33, 4·77	0·90	0·79	0·80	0·43
Leg lean mass	15·02	13·69, 16·49	15·47	13·98, 16·89	–0·24	–3·10, 2·62	–1·58	0·80	0·80	0·60
Arm lean mass	5·67	5·19, 6·30	5·90	5·19, 6·74	–0·20	–1·40, 1·00	–3·52	0·85	0·82	0·55
Percentage lean mass, %	66·87	63·65, 71·05	68·52	62·92, 75·22	–1·71	–13·80, 10·38	–2·53	0·75	0·66	0·46
3. Women (*n* 464)										
A. Body FM, kg										
Total FM	26·93	21·42, 32·32	27·30	20·15, 33·55	–0·04	–7·68, 7·60	–0·15	0·92	0·92	0·69
Truncal FM	12·94	9·82, 16·18	14·35	10·40, 17·40	–1·05	–5·32, 3·22	–8·03	0·91	0·87	0·69
Leg FM	9·04	7·22, 10·80	7·40	5·80, 9·20	1·64	–1·83, 5·11	17·61	0·78	0·68	0·42
Arm FM	3·67	2·80, 4·59	4·00	2·70, 5·50	–0·54	–3·07. 1·99	–14·13	0·88	0·75	0·62
Percentage body fat, %	42·27	38·29, 45·80	41·55	35·30, 46·85	1·17	–9·10, 11·44	2·81	0·82	0·73	0·54
B. Lean body mass, kg										
Total lean mass	36·27	33·40, 40·11	37·50	34·80, 41·50	–1·36	–8·08, 5·36	–3·68	0·81	0·77	0·62
Truncal lean mass	18·58	16·88, 20·79	18·35	16·80, 20·40	0·43	–3·37, 4·23	2·26	0·76	0·74	0·51
Leg lean mass	10·57	9·66, 11·81	10·90	9·89, 12·18	–0·24	–2·93, 2·45	–2·23	0·79	0·73	0·58
Arm lean mass	3·70	3·35, 4·25	4·11	3·61, 4·74	–0·35	–1·27, 0·57	–9·23	0·79	0·66	0·57
Percentage lean mass, %	57·17	53·50, 61·38	58·45	53·19, 64·67	–1·77	–12·2, 8·66	–3·06	0·81	0·72	0·52

Data are presented as median (IQR).

* Difference was calculated by [DXA value-in-body BIA value].

† Percentage of difference (%) was calculated by [ 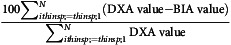 ].

‡ *P* < 0·001.

§ Kappa coefficient was calculated by variable values categorised into trisection.

**Fig. 1:**
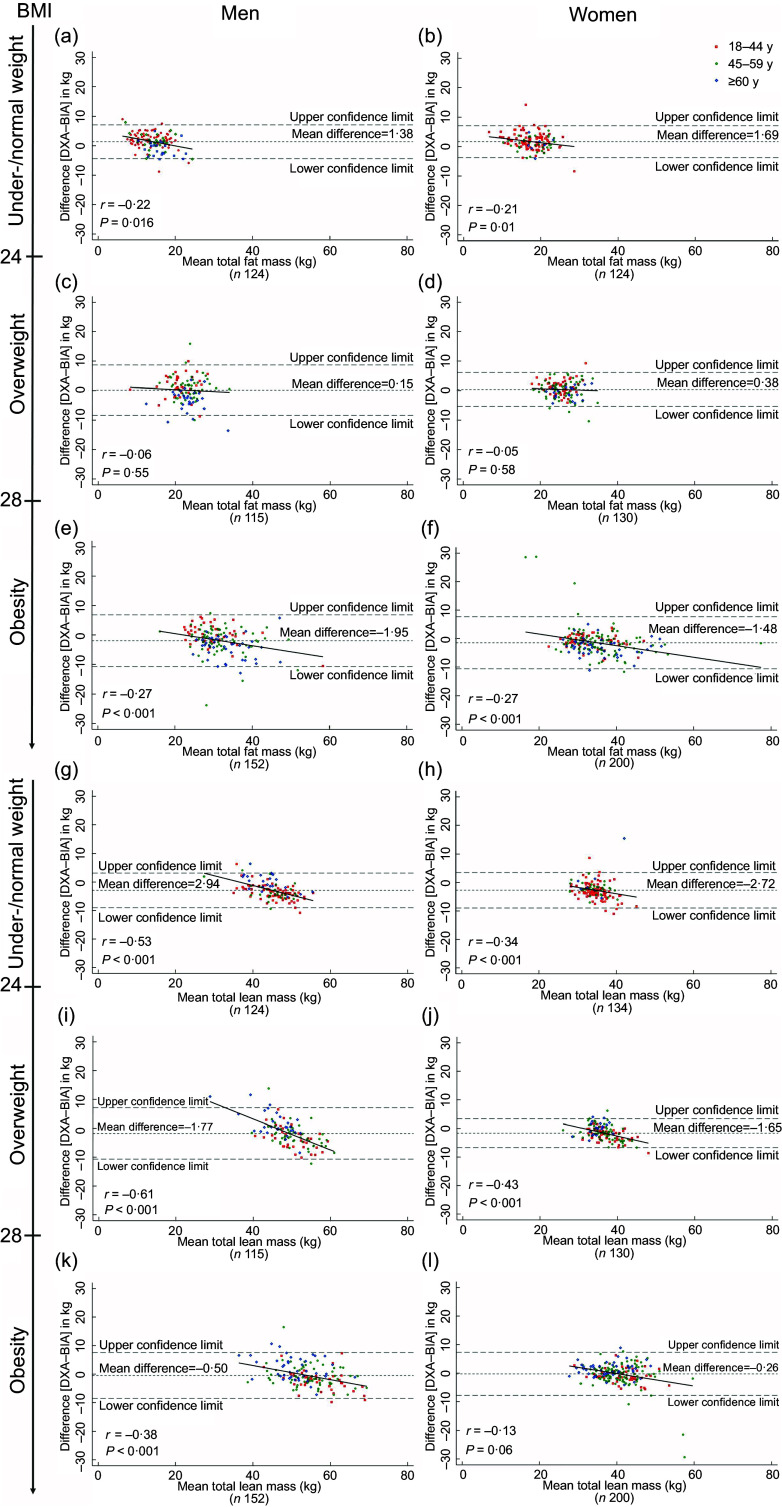
Bland–Altman plots for the comparison of total fat mass and total lean mass measured by dual-energy X-ray absorptiometry (DXA) and bioelectrical impedance analysis (in-body BIA) in Tibetan adults across BMI and sex. Values were obtained from 855 participants. Correlation coefficients derived from Spearman’s correlation. Individuals from different age groups, 18–44, 45–59 and ≥60 y, were represented by red, green and blue points, respectively.

In the Bland–Altman analysis, with respect to total FM, the mean differences between the DXA *v*. the in-body BIA values in under-/normal weight group were 1·38 kg (limits of agreement: –4·25, 7·01) and 1·69 kg (limits of agreement: –3·62, 7·00) in men and women, respectively. Assessment of bias shows that, compared with DXA, in-body BIA seemed to underestimate total FM at lower levels and overestimate it with higher levels of total FM in under-/normal weight group (men, *P* = 0·016; women, *P* = 0·01) (Fig. 1(a) and (b)). The corresponding mean difference values in overweight group were 0·15 kg (–8·22, 8·52) and 0·38 kg (–5·23, 5·99), and differences between the estimates of total FM were not associated with the amount of fat (*P* = 0·55 and *P* = 0·58, respectively) (Fig. 1(c) and (d)). For obese men and women, mean differences between the two methods were –1·95 kg (limits of agreement: –10·57, 6·67) and –1·48 kg (limits of agreement: –10·44, 7·48), with significant bias (*P* < 0·001) observed (Fig. 1(e) and (f)). By contrast, in-body BIA gave lower mean values of total LM in all groups, Spearman’s correlation coefficients between the average total LM and the difference between methods in total LM estimate were significant, except for obese women (Fig. 1(g)–(l)). Absolute limits of agreement of DXA with in-body BIA were wide, particularly for total FM in obese men and women and for total LM in overweight men (Fig. 1(e), (f) and (i)).

### Distribution characteristics of body composition

**Fig. 2:**
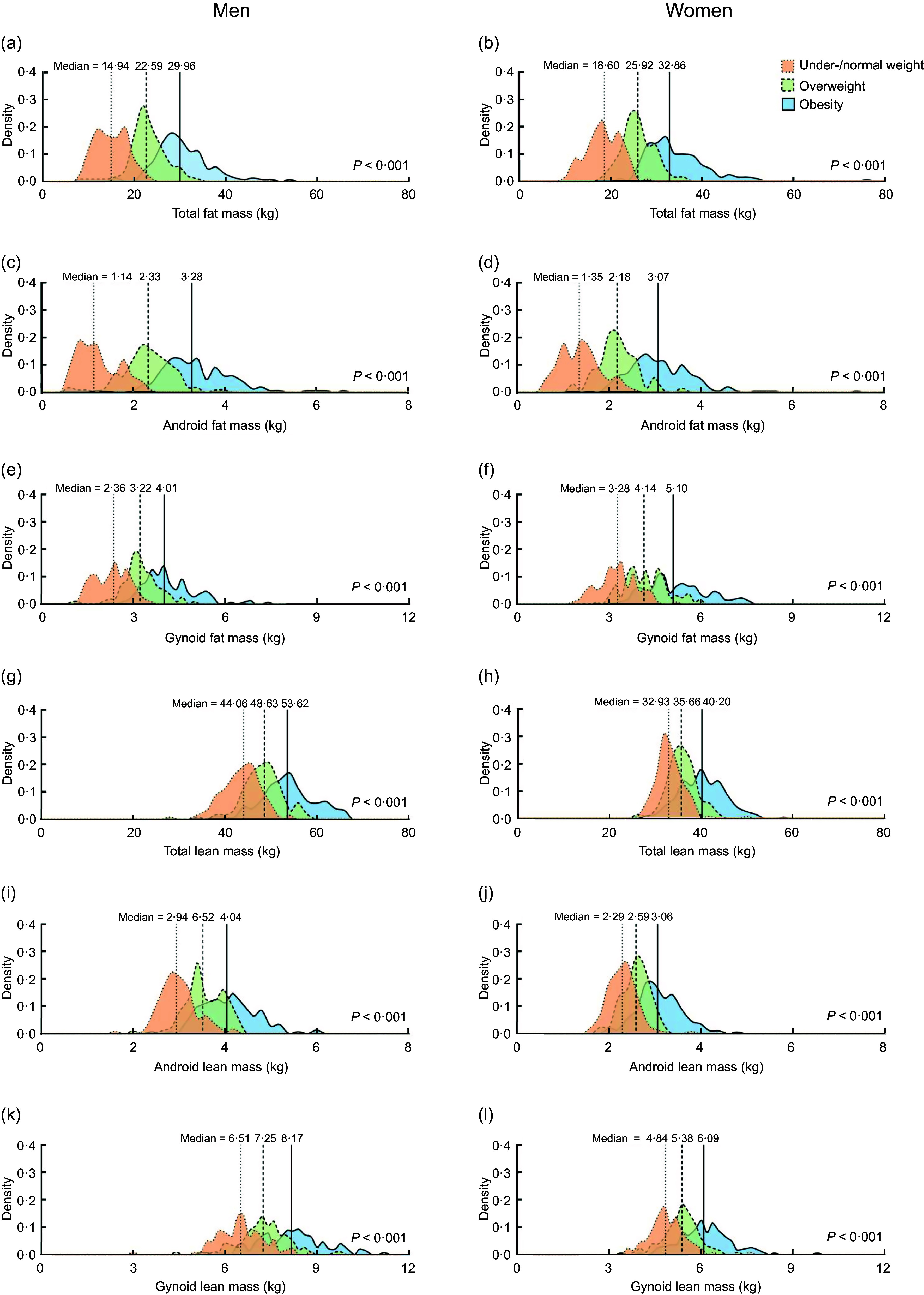
Density plots for body fat mass and lean body mass in Tibetan adults stratified by sex and BMI. Values were obtained by dual-energy X-ray absorptiometry (DXA) from 855 participants. Kruskal–Wallis *H* test was performed to compare variables across BMI groups.

The density plots (Fig. 2) compare FM and LM in total and in the android and gynoid regions. Median values of the six measures assessed by DXA were substantially different in subjects with the three BMI categories within the same sex (*P* < 0·001), and when BMI was high, high BC measured can be observed (Fig. 2). The median total FM values in obese men and women were 29·96 *v*. 32·86 kg, whereas the corresponding median android FM were 3·28 and 3·07 kg, respectively (Fig. 2(a)–(d)).

**Fig. 3:**
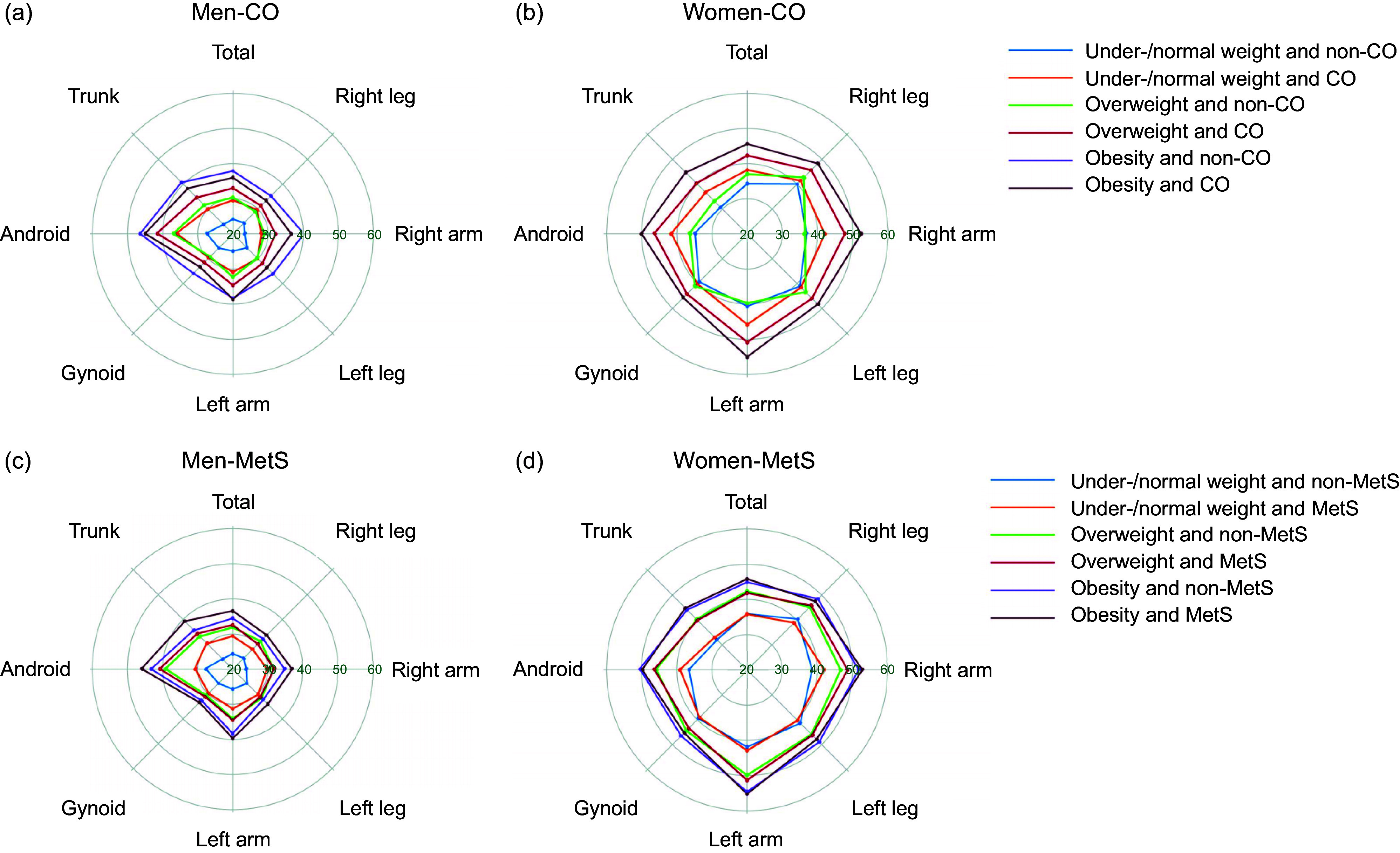
Body fat profiling of Tibetan adults based on sex, BMI categories and metabolic health conditions. Median percentages of fat in total and seven body regions were obtained by dual-energy X-ray absorptiometry (DXA) from 855 participants. Central obesity was defined as waist circumference ≥90 cm for men or ≥80 cm for women. Metabolic syndrome was defined if ≥3 criteria were fulfilled: (1) central obesity; (2) fasting plasma glucose ≥ 5·6 mmol/l or on medication for high blood glucose; (3) systolic blood pressure ≥130 mmHg or diastolic blood pressure ≥85 mmHg or on antihypertensive medication; (4) HDL cholesterol (HDL-C) <1·03 mmol/l for men and <1·30 mmol/l for women or on medication for reduced HDL-C; (5) TAG ≥ 1·7 mmol/l or on medication for elevated TAG. Independent *t*-test or Mann–Whitney *U* test was used for comparison between subjects with and without central obesity or metabolic syndrome. CO, central obesity; MetS, metabolic syndrome.

DXA derived median %BF of Tibetan adults with different BMI and metabolic disorders by sex are displayed in Fig. 3. The dominant %BF was obtained from android region in men regardless of BMI, CO and MetS, but the most noticeable %BF in women was derived from limbs, where the leading one changed with BMI and metabolic status. Among obese men and women with CO, median %BF in android region was high at 44·89 % (*n* 150) and 49·96 % (*n* 200), respectively, whereas median %BF in left and right arm was > 50 % in women and < 40 % in men (Fig. 3(a) and (b)). For overweight men, there was a notable difference in the eight %BF variables between participants with CO (*n* 85) and those without (*n* 30) (*P* < 0·01) (Fig. 3(a) and (b)). Percentages of total FM, android FM, trunk FM, left and right arm FM were also markedly different between women with (*n* 41) and without (*n* 92) CO in under-/normal weight group (*P* < 0·001). When comparing groups with and without MetS, difference in total FM (*P* = 0·002) and trunk FM (*P* < 0·001) proportion were detected, and the median %BF in android region among obese men was 45·77 % (*n* 105) and 43·19 % (*n* 41), respectively (*P* = 0·02) (Fig. 3(c) and (d)). Although no remarkable difference in android %BF was found among obese women, the gynoid %BF (MetS, *n* 122, mean = 45·23 ± 3·61; non-MetS, *n* 69, mean = 46·60 ± 3·58 kg) was significantly different (*P* = 0·012). Additionally, right arm %BF in overweight women (MetS, *n* 47, mean = 48·63 ± 4·80; non-MetS, *n* 77, mean = 46·67 ± 5·10 kg) was significantly different (*P* = 0·036).

## Discussion

In this study, we reported for the first time the validity of In-Body BIA to assess BC in a Tibetan adult population in Qinghai, China, by using DXA as reference. Our results suggest that in-body BIA assessments of BC provided good relative agreement with DXA, as revealed by high correlation coefficients (Spearman’s r and Lin ρ). In absolute terms, in-body BIA tended to overestimate total FM, total LM and total LM proportion and underestimate total FM proportion compared with DXA. We also described the BC profiling in participants with different BMI and metabolic status.

The relative agreement with DXA for BC assessed by in-body BIA as continuous variables was generally satisfactory or good in Tibetan adults^(24,25)^. This finding is in accordance with prior studies, which have reported high correlations between DXA and in-body BIA^(26,27)^. Nevertheless, mediocre Lin ρ were observed in women for leg FM and arm LM and in men for percentage total LM. When evaluating the correlation of BC trisection by DXA and In-Body BIA, we found moderate to substantial agreement. The total FM and total LM generally showed better relative agreement than regional BC measures in men, women and all participants, with total FM demonstrating the highest correlation coefficients.

In all participants, the percentage of bias for the absolute difference between in-body BIA and DXA were between 0·58 and 14·11 % for the ten tested variables including both percentage of BC mass and absolute value (kg); and the in-body BIA overestimated body FM and LM compared with DXA results except leg FM, total FM proportion and truncal LM. Previous findings in Canadian adults reported a bias from 8 to 11 % using In-Body BIA^(28)^. Mean differences between DXA and in-body BIA were approximately 14–15 % in FM and %BF in Finnish women and men^(29)^.

Despite reporting generally low bias, the wide absolute limits of agreement of DXA with in-body BIA regarding total FM and total LM demonstrated the limitation of the use of in-body BIA-based BC values at the individual level. These wide limits of agreement are in line with prior reports^(30)^, which may reflect an intrinsic problem with in-body BIA, and larger absolute limits of agreement were noted in obese subjects and overweight men compared with overweight women as well as under-/normal weight individuals. Among Tibetan adults, there was a tendency that the absolute difference value of [DXA-in-body BIA] grew with the increase of total FM and total LM, with significant correlations between the bias and measurement averages in most BMI categories by sex. A comparison between fat-free mass values assessed by DXA and in-body BIA in healthy Chinese men and women (*n* 554; age range, 16–75 years) from Taiwan reported small systematic error, and the absolute limits of agreement of Bland–Altman analysis was (–6·40, 6·40) kg^(31)^. Another study among Chinese children from Beijing showed that in-body BIA significantly estimated a lower fat content (bias = 2·5 kg in boys and bias = 2·7 kg in girls) but higher fat-free mass (bias = 1·8 kg in boys and bias = 2·9 kg in girls) than DXA^(32)^. Previous research comparing in-body BIA and DXA, which included Frenchmen and Mexican, implicated an overestimation of lean body mass and underestimation of FM using in-body BIA^(7,33)^, but some other studies showed inverse results^(34,35)^. The present study provided evidence across BMI categories, lifespan and sex that in-body BIA overestimated total LM in all subjects and total FM in overweight as well as obese subjects, whereas it underestimated total FM in under-/normal weight ones. Accordingly, it revealed that the prior controversial conclusions could be partly explained by demographic heterogeneity, yet deserve further investigation.

The systematic errors between DXA and in-body BIA might be in part due to differences in hydration status that emerge with varying levels of BF. Studies have noted that total body water and relative extracellular water are greater in individuals with obesity compared with those with normal weight^(36)^. As DXA is less sensitive than in-body BIA to differences in hydration^(37)^, it could be expected that this would affect the agreement between the two methods at various BF levels. On the other hand, the bias between the assessment of the two methods may be attributed to the algorithm used in in-body to estimate BC or variation in body geometry among different ethnic groups^(29)^. It is also important to note that our results are applicable only to the in-body BIA device, and results from other BIA devices may differ.

It is noteworthy that within the same sex and BMI category, individual BF profiling distinction existed in Tibetan adults, combined with divergent phenotypes of metabolic status. A study conducted in non-Hispanic Caucasian claimed that body FM and BF distribution are more sensitive than BMI in identifying cardiometabolic risk^(2)^. The present study to some extent confirms it and highlights the importance of investigating associations between adiposity and cardiometabolic disorders in the Tibetan population. It will be of value in metabolic health management, especially for those with normal weight but potentially high risk of cardiometabolic diseases. Future studies focusing on the diversity in disease associations to multivariable BC to explain the complex picture^(38)^ are warranted. Moreover, BF changes independent of BMI may be considered to serve as proxies of cardiometabolic benefits of a given intervention^(39)^.

The Tibetan population, as the native highlanders on the Qinghai-Tibetan Plateau, seems to have a distinctive body fat distribution from non-highlanders. More specifically, Tibetans tended to have higher FM percentage compared with other non-highlander populations when their BMI were comparable or even lower than other populations, such as White, Black and Han populations in China^(40–42)^. When BMI was similar, the Tibetan population in this study had a 6–8 % higher body fat percentage than the Han population (men, 32·2 % *v*. 24·3 %; women, 42·3 % *v*. 36·3 %)^(42)^. Further, adiposity tended to accumulate in the abdomen for Tibetan, shown as a larger difference in the gap between android FM percentage and other body parts in Tibetans in our study than in participants in the NHANES study^(40)^. This may be related to the adaptation to the extreme cold climate in high-altitude areas, where mammals tend to have more fat reserves to maintain thermoregulation^(43)^. It is also hypothesised that the Tibetan population, who have ancestral exposure to long-term cold, probably have more brown adipose tissue (BAT) and enhanced BAT thermogenesis from an evolutionary perspective^(44)^. This hypothesis is supported by evidence from native mammals exposed to chronic cold on the Qinghai-Tibetan Plateau, in which subcutaneous adipose tissue browning and altered global metabolism have been observed^(45)^. This hypothesis of BAT-induced thermogenesis and excess calorie burning will also help explain the relatively low prevalence of obesity defined by BMI in the Tibetan population, as mentioned in the introduction section.

Our study has several strengths. It is the first one to assess the validity of In-Body BIA with reference to DXA in a large sample of Tibetan adults who live in the Tibetan Plateau. In addition, we investigated the characteristics of BC in the population, which may help to uncover the impacts of the special environment on BC and the link with cardiometabolic consequences in high-altitude zones. Moreover, participants have lived in the surveyed communities for at least 3 years; this long-term residence enables a more accurate assessment of environmental impacts, reducing data bias caused by short-term residents and thereby enhancing the reliability and validity of the study results. Limitations of this study include the absence of consideration for the hydration status of the examined population, despite the established influence of hydration on in-body BIA outcomes^(46)^. Additionally, the cross-sectional design of the study solely depicts the observed association between BC and metabolic status rather than causality. It is also important to note that our results are applicable only to the in-body BIA device, and results from other BIA devices may differ. Moreover, participant dropout due to missing data—particularly related to conducting DXA measurements in a challenging high-altitude environment—could affect both the internal and external validity of the study. While our findings may not be fully generalisable to other populations, they align with those of similar studies, supporting external validity. In terms of internal validity, our study provides statistically significant results within this unique population; however, further research is needed to strengthen these findings.

## Conclusions

In-body BIA is a reliable method for assessing body FM and LM at the group level referenced by DXA in the Tibetan population, but the two methods for individual BC measurement may not be interchangeable in the clinical setting. Although the differences at the group level are acceptable, there are substantial individual differences that need to be considered. Further, the Tibetan population tended to have more FM compared with non-highlanders with comparable BMI levels. Gradience in the distribution of total and regional FM content was observed across different BMI categories and its combinations with waist circumference and metabolic status.

## Data Availability

Some or all datasets generated during and/or analysed during the current study are not publicly available but are available from the corresponding author on reasonable request.
